# Reply to: Indeterminate Ultrasonography Should Not be Reclassified as a Negative Test in Patency Capsule Localization

**DOI:** 10.1002/deo2.70354

**Published:** 2026-05-19

**Authors:** Tomoharu Matsuura, Satoshi Osawa

**Affiliations:** ^1^ Department of Laboratory Medicine Hamamatsu University School of Medicine Shizuoka Japan; ^2^ Department of Advanced Medical Science for Regional Collaboration, Hamamatsu University School of Medicine Shizuoka Japan; ^3^ Department of Endoscopic and Photodynamic Medicine, Hamamatsu University School of Medicine Shizuoka Japan

To the Editor,

We thank Koulaouzidis and Toth [1] for their thoughtful and constructive comments on our recent publication [2]. We fully agree that non‐visualized ultrasonographic findings should be interpreted as indeterminate rather than negative, and we appreciate the opportunity to clarify this important point.

In our study, the binary classification used for the primary endpoint was explicitly defined as an operational framework for statistical analysis and was not intended to imply that “not visualized” findings represent true negative results. As correctly highlighted by the authors, we stated in the manuscript that non‐visualization does not exclude small‐bowel retention and should prompt further evaluation, typically with computed tomography.

Importantly, we also performed a three‐category analysis (“small bowel,” “colon,” and “not visualized”) to reflect real‐world clinical interpretation, in which non‐visualized cases were treated as indeterminate. In this context, abdominal ultrasonography (AUS) provided definitive localization in 82.4% of patients, which we consider to represent the clinically relevant performance of the modality. Similar diagnostic patterns have been reported in previous studies, with visualization rates of 74.5% (13/17) in Shiotani et al. [3] and 82.5% (47/57) in Handa et al. [4].

We also acknowledge the limitations of our study, including the small number of small‐bowel retention cases and the potential for incorporation bias, as discussed in the manuscript. These factors warrant cautious interpretation and further validation in larger, multicenter studies.

In summary, we share the authors’ view that AUS should be considered a triage tool rather than a definitive stand‐alone test, and that indeterminate findings appropriately prompt additional imaging. Our results support the role of AUS as a radiation‐free first‐line modality within this clinical framework.

We appreciate the authors’ insightful comments, which further emphasize the importance of careful interpretation and appropriate clinical application of ultrasonography in patency capsule assessment.

## Author Contributions

Tomoharu Matsuura and Satoshi Osawa contributed equally to the drafting and revision of the manuscript and approved the final version.

## Conflicts of Interest

The authors declare no conflicts of interest.

## Funding

The authors have nothing to report.

## Artificial Intelligence Disclosure

No artificial intelligence tools were used in the preparation of this manuscript.
